# Using oriented peptide array libraries to evaluate methylarginine-specific antibodies and arginine methyltransferase substrate motifs

**DOI:** 10.1038/srep28718

**Published:** 2016-06-24

**Authors:** Sitaram Gayatri, Martis W. Cowles, Vidyasiri Vemulapalli, Donghang Cheng, Zu-Wen Sun, Mark T. Bedford

**Affiliations:** 1 Department of Epigenetics and Molecular Carcinogenesis, The University of Texas MD Anderson Cancer Center, Smithville, TX 78957, USA; 2 Epicypher Inc., Research Triangle Park, NC 27709, USA; 3 Graduate Program in EMC, UT GSBS, Houston, Texas 77030, USA

## Abstract

Signal transduction in response to stimuli relies on the generation of cascades of posttranslational modifications that promote protein-protein interactions and facilitate the assembly of distinct signaling complexes. Arginine methylation is one such modification, which is catalyzed by a family of nine protein arginine methyltransferases, or PRMTs. Elucidating the substrate specificity of each PRMT will promote a better understanding of which signaling networks these enzymes contribute to. Although many PRMT substrates have been identified, and their methylation sites mapped, the optimal target motif for each of the nine PRMTs has not been systematically addressed. Here we describe the use of Oriented Peptide Array Libraries (OPALs) to methodically dissect the preferred methylation motifs for three of these enzymes – PRMT1, CARM1 and PRMT9. In parallel, we show that an OPAL platform with a fixed methylarginine residue can be used to validate the methyl-specific and sequence-specific properties of antibodies that have been generated against different PRMT substrates, and can also be used to confirm the pan nature of some methylarginine-specific antibodies.

Posttranslational modifications (PTMs) on proteins drive signal transduction from the cell surface into the nucleus, thus allowing cells to react to extracellular stimuli. This process of signal transduction is often deregulated in disease states, especially cancer. Central to these signaling pathways are specific PTMs, including phosphorylation, acetylation and methylation. Arginine methylation was first reported in the 1960s^1^, but only recently has its biological functions started to be realized^2^,^3^. Arginine methylation is an abundant PTM, with about 0.5% of arginine residues methylated in mammalian tissues^4^. Most (>60%) of the arginine methylated residues are found in proteins that associate with RNA^5^. However, there is also a clear role for this PTM in histone methylation and epigenetic signaling^6^.

There are nine mammalian protein arginine methyltransferases (PRMTs)^7^. These enzymes catalyze three types of arginine methylation: ω-N^G^-monomethylarginine (MMA), ω-N^G^,N^G^-asymmetric dimethylarginine (ADMA) and ω-N^G^,N′^G^-symmetric dimethylarginine (SDMA). The majority of the PRMTs methylate glycine- and arginine-rich (GAR) motifs within their substrates, which can be methylated in both an ADMA and SDMA fashion^8^. The outlier is CARM1, which displays unique substrate specificity in that it does not methylate GAR motifs^9^, but rather a PGM motif, which is proline- and glycine-rich^10^. PRMT5 can also symmetrically dimethylate arginine residues within a subset of PGM motifs^10^. Mass spectrometric analysis of arginine methylation sites has identified substrates that do not adhere to the rather loose definition of PGM and GAR motifs^11^,^12^, indicating that methylation motifs for the different PRMTs have yet to be well defined.

To address this issue, we adapted a synthetic peptide combinatorial library approach to interrogate PRMT methylation motifs in an unbiased manner and identify the linear sequences recognized by methylarginine-specific antibodies. This approach was first proposed by Richard Houghten^13^, and then further developed by the Cantley laboratory for the analysis of SH2 binding motifs^14^ and the identification of optimal substrate sequences for protein kinases^15^. In this approach, soluble pools of random peptides are oriented relative to a central “fixed” amino acid, which could be a tyrosine or phospho-tyrosine residue as in the Cantley studies highlighted above, but in our case will be an arginine or methylarginine residue. This orienting of the peptides around a fixed residue restricts the degeneracy of the library and prevents the PRMT methylation motif or antibody-binding motif from moving out of register. The peptides that bind a domain or antibody, or those modified by an enzyme of interest are then enriched and subjected to Edman degradation sequencing as a mixture to deconvolute the consensus motif. To make peptide sequencing unnecessary, this approach is again modified to now incorporate a scan sub-library strategy, along with the printing of the sub-libraries onto glass slides^16^. This platform has been termed an oriented peptide array library, or OPAL. This OPAL platform has been successfully used to identify SH2 binding motif^16^,^17^,^18^, to determine kinase phosphorylation specificity^19^, and to identify short linear epitopes that are recognized by phospho-specific antibodies^16^. Here we learn from the use of OPALs in the phosphorylation field, and apply this technology to the study of arginine methylation.

## Results

### Epitope Mapping of Methylarginine-specific Antibodies Using an OPAL

#### Validating the Methyl-specific Nature of the Antibodies to be Tested

**Figure 1:**
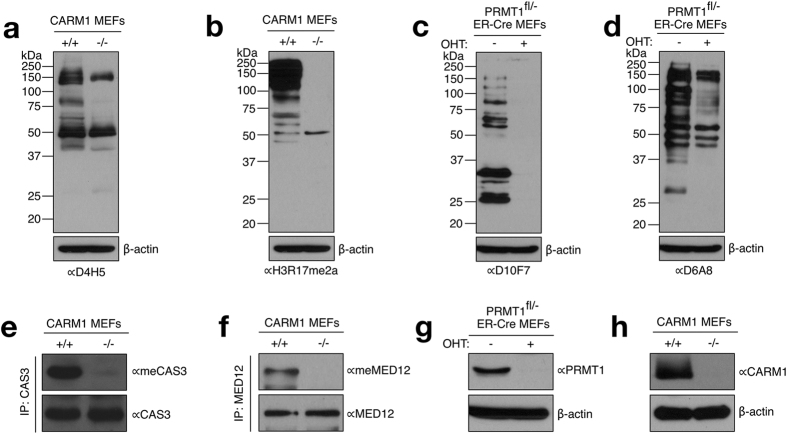
Characterization of methylarginine-specific antibodies. The antibodies to be used on the OPAL platform were first tested to establish that they are indeed methylarginine specific. (**a,b**) Whole cell extracts for CARM1 wild-type (+/+) and knockout (−/−) MEFs were subjected to Western analysis with αD4H5 and αH3R17me2a antibodies. (**c,d**) PRMT1^fl/−^ ER-Cre MEFs were untreated or treated with 2 μM 4-hydroxytamoxifen (OHT) for 8 days and subjected to Western analysis using αD10F7 and αD6A8 antibodies. (**e,f**) CARM1 wild-type (+/+) and knockout (−/−) MEF extracts were immunoprecipitated (IPed) using non-methyl specific antibodies against CAS3 and MED12. Western analysis was then performed, with CAS3 and MED12 antibodies that were used for the IP to ensure equal expression in both cell lines, and then with meCAS3 and meMED12 antibodies to detect the methylated forms of the proteins. (**g,h**) Western analysis was performed with αCARM1 and αPRMT1 antibodies to confirm the knockout of these PRMTs in the lysates used for the studies in this figure.

We have gathered eight different Rme2a-specific antibodies to test on the OPAL platform; three of these antibodies were generated against a redundant XXRme2aXX antigen (D10F7, D6A8 and D4H5) with the expectation of producing pan-ADMA antibodies^20^, one antibody that was raised against the H3R17me2a mark but has been shown to cross react with a large number of CARM1 substrates^10^, and four ostensibly specific antibodies (H3R2me2a, H3R8me2a, CAS3R87me2a & Med12R1899me2a). By Western blot analysis, using PRMT1 or CARM1 knockout cells, we demonstrated that the D10F7, D6A8, D4H5 and H3R17me2a antibodies recognize a number of PRMT substrates, as expected (Fig. 1a–d). We also showed that the antibodies raised against CARM1 substrates CAS3R87me2a and Med12R1899me2a, which we recently identified (unpublished data), require CARM1 for immunoreactivity (Fig. 1e,f). Both PRMT1 and CARM1 are efficiently knocked-out in the lysates that were used for the analysis (Fig. 1g,h). The histone code antibodies against the H3R2me2a and H3R8me2a marks are commercially available from Millipore and were not retested here. All eight of these antibodies were further evaluated using Rme2a-fixed OPAL.

#### Designing the Fixed-methylarginine (Rme2a) Oriented Peptide Array Library

**Figure 2:**
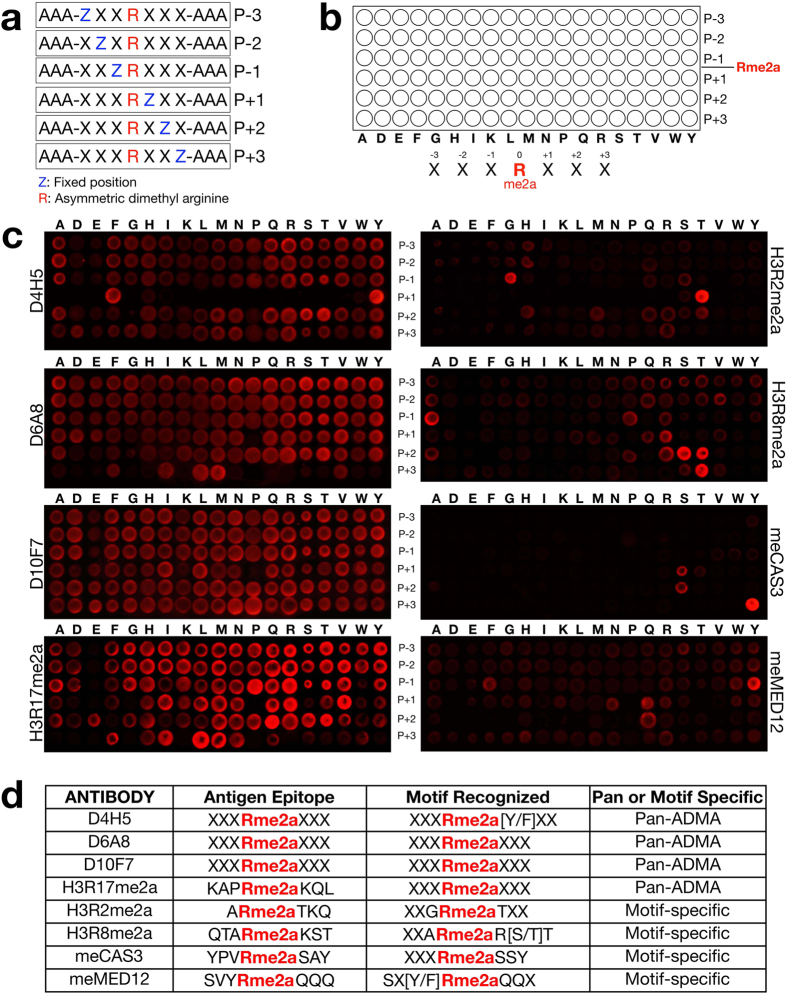
Preference of methyl-specific antibodies determined by OPAL. (**a**) Design of the peptide library for determining the recognition motifs of methyl-specific antibodies. (**b**) Design of the Rme2a-OPAL once arrayed. (**c**) The Rme2a-OPAL was probed with pan-ADMA antibodies (D4H5, D6A8 and D10F7) and with antibodies that were raised against a specific methyl-motif (H3R17me2a, H3R2me2a, H3R8me2a, CAS3R87me2a (meCAS3) and MED12R1899me2a (meMED12)). (**d**) A list of the eight antibodies used to probe the OPAL, the antigen epitope used to generate the particular antibody, and the motif that is recognized on the OPAL.

An oriented library of thirteen amino acids (AAAXXX**Rme2a**XXXAAA) was synthesized with methylarginine (Rme2a) fixed in the middle of the peptide and in which X is random (any amino acid except cysteine). The peptide was designed with three alanine (A) residues at both the C- and N-termini, which bracket the redundant residue positions in the middle. We chose to synthesize a peptide library with three redundant positions (X) on either side of the fixed methylarginine residue, because the average antibody linear epitope size falls into this range^21^, and previous OPAL studies of phospho-specific antibodies identified epitope motifs that straddle the fixed-phosphotyrosine residue by up to three residues^16^. The peptides were derivatized at their C-termini for covalent attachment to glass slides. For screening, the relative importance of different amino acids at defined positions is determined by a scanning strategy. This strategy is depicted in Fig. 2a. For example, with position P-3 fixed (**Z**) in the AAA**Z**XX**Rme2a**XXXAAA library, 19 different libraries were synthesized, and each library has one of the 19 amino acids fixed at position “**Z**”. For the next row, the “**Z**” position moves to P-2 (two residues N-terminal from the fixed Rme2a), and again 19 different libraries are synthesized. A total of 114 (19 amino acids × 6 positions) independent library pools were synthesized. The 114 different peptide libraries were synthesized and arrayed onto glass slides by PepScan (Lelystad, The Netherlands) (Fig. 2b). In this manner, the pattern of antibody recognition can then be simply read off the arrays and converted into a consensus recognition motif.

#### Screening the Rme2a-fixed OPAL with Methyl-specific Antibodies

Both basic research laboratories and commercial entities have developed methyl-specific antibodies. However, it has been difficult to gauge the quality of these reagents. Here we used several pan and sequence-specific ADMA antibodies, which we have validated on lysates from knockout cell lines (Fig. 1), to test the ability of the Rme2a-OPAL platform to facilitate rapid and unbiased epitope characterization. The antibodies that recognize a large number of proteins in a methyl-dependent manner (D10F7, D6A8, D4H5 and H3R17me2a), as demonstrated by Western analysis, displayed limited pattern recognition on the Rme2a-OPAL, validating their pan-ADMA properties (Fig. 2c). Interestingly, the D4H5 antibody is generally pan-ADMA, however at the P+1 position it selects for F/Y residues. Knowing this information could prove valuable when choosing methyl-specific antibodies to your protein of interest.

In comparison to the pan-ADMA antibodies, the antibodies that have been generated to a specific sequence (H3R2me2a, H3R8me2a, CAS3R87me2a & MED12R1899me2a), recognized a very restricted motif on the Rme2a-OPAL (Fig. 2c). More importantly, the sequence used as the antigen for producing the antibody is very similar to the motif that the antibody recognizes on the Rme2a-OPAL (Fig. 2d). For example, the αMED12R1899me2a antibody was raised against a SVY**Rme2a**QQQ peptide. The OPAL experiment reveals that the motif recognized by this antibody is – SXF/Y**Rme2a**QQX, which is perfectly nested in the antigen sequence (Fig. 2c,d). The αH3R8me2a antibody was raised against a QTA**Rme2a**KST peptide, and the unbiased OPAL experiment shows that it recognizes an XXA**Rme2a**RS/TT motif that is very similar to the antigen (Fig. 2c,d). Likewise, the αH3R2me2a antibody that was raised against a A**Rme2a**TKQ peptide has recognition motif of XXG**Rme2a**TXX, and the αCAS3R87me2a antibody that was raised against a YPV**Rme2a**SAY peptide has recognition motif of XXX**Rme2a**SSY.

### *In Vitro* Methylation by PRMTs of Oriented Peptide Libraries

#### Designing the Fixed-arginine (R) Oriented Peptide Array Library

**Figure 3:**
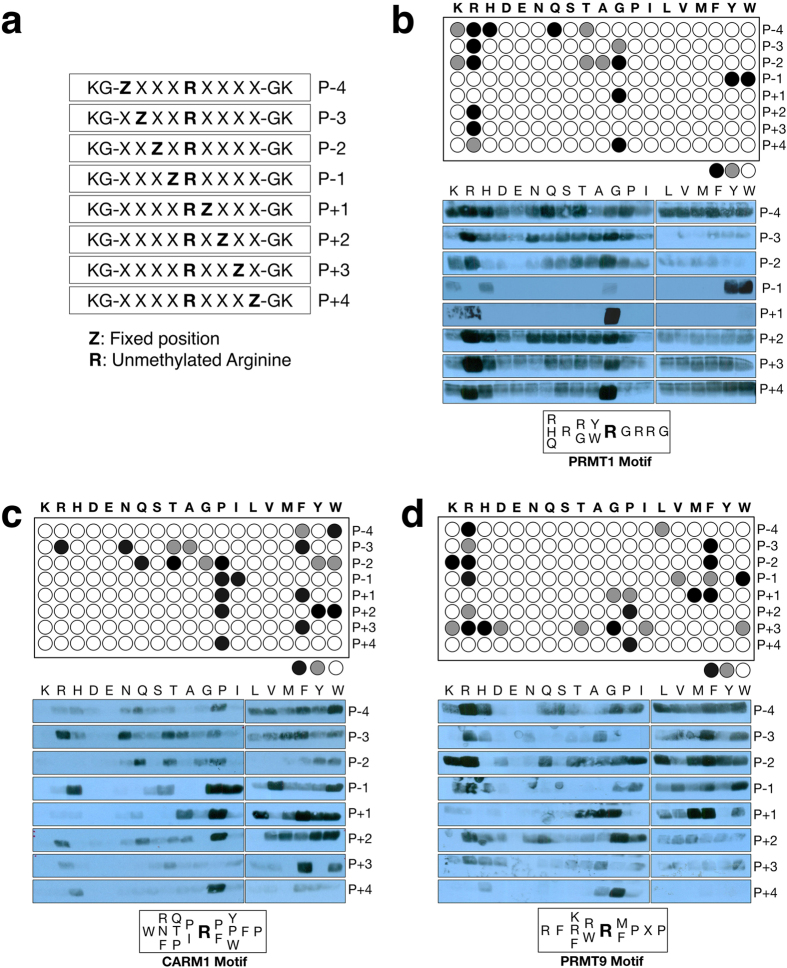
Substrate specificity of PRMT1, CARM1 and PRMT9. (**a**) Design of the peptide library for determining the methylation motifs of PRMTs. (**b–d**) *In vitro* methylation assays were performed on each of the 152 peptide pools, and these methylated peptides were then subjected to SDS-PAGE and exposed to film to detect the peptide pool that could be methylated by each enzyme (middle panels). The signal intensity of each pool was gauged by densitometry and depicted in an array format (top panels), with the black circle representing strong signals (70% and above, relative to the strongest signal in each row), the gray circles representing medium intensity signals (50–69%), and the open circle representing weak or no signals. The motifs for three PRMTs (PRMT1 (**b**), CARM1 (**c**) and PRMT9 (**d**)) were deciphered from the arrays and are presented (bottom panels).

To perform *in vitro* methylation reactions on oriented peptide libraries, an unmodified arginine needs to be fixed in the central position. In addition, we enlarged the library to now have four redundant positions (X) on either side of the fixed arginine residue, because mass spectrometry studies suggest that the fourth position after the arginine residue may be important for certain unidentified PRMTs^12^. A total of 152 (19 amino acids × 8 positions) independent library pools were synthesized and arrayed onto glass slides by PepScan (Fig. 3a).

#### Screening the R-fixed OPAL with PRMTs

We chose to focus on three different arginine methyltransferases, PRMT1, CARM1 (PRMT4) and PRMT9, which would likely have very distinct methylation motifs. PRMT1 is known to selectively methylate glycine/arginine-rich (GAR) motifs, CARM1 methylates proline/arginine-rich (PGM) motifs, and the recently characterized PRMT9 has a single identified substrate (SAP145) and cannot methylate either GAR or PGM motifs^22^. R-fixed OPALs containing an unmodified arginine in the fixed position were incubated with recombinant PRMT1, PRMT9 and CARM1, and radiolabeled AdoMet. As positive controls, a known substrate for each PRMT was also included in a parallel experiment to demonstrate that these enzymes are indeed active in an *in vitro* methylation assay (histone H3 for CARM1, SAP145 for PRMT9, and histone H4 for PRMT1). After *in vitro* methylation, the arrays were washed, sprayed with enhance and subjected to fluorography. Unfortunately, even after a very long exposure (21 days) no radioactive signal was observed on these arrays, suggesting that there was not enough substrate present on the array to allow the detection of the rather weak tritium signal.

We thus altered our strategy and performed these experiments in a soluble format. For this “brute force” approach, we went back to the 152 library pools of soluble peptides, and 1 μg of each library was incubated with 1 μg of enzyme and radio-labeled AdoMet. These *in vitro* methylated peptides were then resolved by SDS-PAGE and exposed overnight to obtain the observed signals (Fig. 3b–d). The signal intensities were then converted into an array format for easy interpretation, and the optimal methylation motif for each enzyme was identified. Very different and distinct methylation patterns were observed for each of the three PRMTs: a core RG or GAR motif for PRMT1 with selectivity for Y/W at P-1, a clear preference for a proline residue near the fixed arginine residues for CARM1 (PRMT4), and a strong preference for charged R/K/F residues at P-2 and M/F at P+1 for PRMT9. Similarly to CARM1, PRMT9 also selects for proline residues at P+2 & +4.

## Discussion

A comprehensive computational analysis of the human proteome has identified 208 proteins that make up the human methyltransferasome^23^, which equates to roughly 1% of all human gene products. The most abundant class of methyltransferases is the seven-β-strand superfamily (with 130 members), which have a Rossmann-like structural core, followed by the SET domain-containing class of methyltransferases (with 56 members). Methyltransferases use a variety of different substrates, including DNA, RNA, small molecules, and proteins. With regards to protein methylation, arginine and lysine residues serve as the primary substrates^24^. Members of both the seven-β-strand and the SET domain-containing superfamilies can methylate proteins. However, many of the proteins identified as potential methyltransferases have yet to be categorized according to their substrate specificity. Using pooled libraries of soluble peptides, with either fixed arginine or lysine residues for *in vitro* methylation reactions, could help rapidly “deorphanize” methyltransferases.

The advantage of OPAL analysis includes: 1) antibody and enzyme motifs are obtained in an unbiased fashion around a fixed central residue, and 2) ease of data interpretation since the motifs can be directly “read” from the array with no need for peptide sequencing. This approach will be particularly powerful in characterizing methyl-specific antibodies, which are otherwise very difficult to gauge. This is particularly concerning for the field of epigenetics, where antibodies of poor quality can lead to misinformed conclusions regarding the location and function of PTMs involved in the histone code. This issue is starting to be addressed with the testing of commercially available histone antibodies on peptide microarrays that harbor histone code related PTMs^25^. By subjecting these antibodies to an OPAL analysis, an additional level of specificity information can be obtained. For example, we have tested three methyl-arginine specific histone code antibodies on the Rme2a-OPAL platform, αH3R17me2a, αH3R2me2a and αH3R8me2a. The αH3R17me2a antibody, which has been reported to recognize many non-histone substrates for CARM1^10^, does indeed have a loose recognition motif (Fig. 2c), although there are stronger signals for proline at the P-1, glutamine at the P+2, and leucine at the P+3 positions. However, the αH3R2me2a and αH3R8me2a antibodies display much more specificity than the αH3R17me2a antibody, and in both cases, their recognition motifs are largely nested within the antigen sequence that was used to generate them (Fig. 2c,d).

Arginine methylation motifs have generally been identified by aligning known substrates for the different PRMTs^10^,^11^,^12^,^26^,^27^, or by using pan methyl-specific antibodies to enrich for methylated peptides that are then identified by mass spectrometry^11^,^28^. The first approach is limited by the relatively small number of specific PRMT substrates that have been identified and mapped, and the second approach is limited by the fact that the methyl-specific antibodies used to enrich for the methylated motifs are likely not totally pan, and could recognize a subset of substrates that would bias the motif description. This second issue has been partially resolved with the antibody-independent enrichment of tryptic peptides that contain methylated arginine residues^12^, and using this approach, two different motifs were unmasked – a glycine/arginine (G/R) motif and proline/arginine (P/R) motif. Here we show, using an oriented peptide library approach, that PRMT1 is likely largely responsible for the G/R methylation, and both CARM1 and PRMT9 could account for the P/R methylation. The observed motif for PRMT1 and CARM1 (Fig. 3b,c) are inline with the reported motifs for these two enzymes^7^,^10^,^29^. Importantly, the crystal structures of CARM1 with three different peptide substrate sequences were recently solved^30^. This is the first example of a type I PRMT structure with defined density for amino acids beyond the substrate arginine residue. All three substrate motifs were proline-rich and it is very clear from the structures that binding interactions allow for the presence of flanking proline residues both N- and C-terminal to the substrate arginine.

Very little is known about the substrate specificity of PRMT9, as SAP145 is the only identified substrate for this enzyme to date. The motif of PRMT9 methylation on SAP145 is – CFK**Rme2s**KYL, which is not at all similar to the motif that we identified using the screen of 152 library pools of soluble peptides (Fig. 3d). It is possible that the cysteine residue at the P-3 position is very important for the methylation of SAP145, and our oriented peptide library does not contain cysteine residues. Alternatively, in the case of SAP145, more than just a linear motif is required for PRMT9 methylation. However, by using the oriented peptide library identified motif for *in silico* screening, it may be possible to identify novel PRMT9 substrates.

## Methods

### Antibodies

D4H5, D6A8 and D10F7 were generated against degenerate peptides containing four asymmetric dimethylarginine (ADMA) residues XXXXRme2aXXRme2aXXXXRme2aXXX Rme2aX by Cell Signaling Technology (CST). D4H5 and D6A8 have been previously described^20^ and are commercially available as part of an ADMA antibody mixture (CST, Catalog #13522). Anti CAS3 antibodies were custom raised by Bethyl laboratories against a peptide pool comprising amino acids 1-20 and 176-195 of human CAS3. CAS3 is also known asFAM168B or MANI; NM_001009993). Anti-methyl CAS3R87me2a (meCAS3) was generated against the epitope CTAVYPVRme2aSAYPQQSR by Covance. Methyl MED12R1899me2a (meMED12) was custom generated against the epitope TSVYRme2aQQQP by CST. PRMT1 antibody was a gift from Stephane Richard (McGill University). H3R2me2a (Millipore, Catalog #05-808), H3R17me2a (Millipore, Catalog #07-214), H3R8me2a (Active Motif, Catalog #39651), CARM1 (Bethyl, Catalog #A300-421A) and β-actin (Sigma, Catalog #A5316) were obtained commercially.

### Cell lines

*Prmt1*^*fl/−*^ ER-Cre MEF line^31^ and *Carm1*^−/−^ MEF line^32^ have been described previously. *Prmt1*^*fl/−*^ ER-Cre MEFs were treated with 2 μM 4-Hydroxytamoxifen (OHT) for 8 days to delete *Prmt1*. Both cell lines were cultured in Dulbecco Modified Eagle Medium (DMEM) with 10% fetal bovine serum.

### Immunoprecipitation and Western blotting

Whole cell extracts were prepared in mild lysis buffer (150 mM NaCl, 5 mM EDTA, 1% Triton X-100 and 10 mM Tris-HCl, pH7.5). *Carm1*^−/−^ and WT MEF lysates were incubated with anti-CAS3 or anti-MED12 antibodies overnight at 4 °C, followed by incubation with Protein A/G beads (Thermo Scientific) for 1 hour. The beads were then washed three times with mild lysis buffer and boiled in loading buffer to elute bound proteins. The immunoprecipitated samples or whole cell extracts were resolved by SDS-PAGE, transferred onto PVDF membranes, and blocked with 5% nonfat dry milk and 0.1% Tween 20 in PBS. Blots were then incubated with appropriate primary antibodies in the blocking buffer overnight at 4 °C. The blots were then washed and probed with HRP-labeled secondary antibody and detected using chemiluminescence (Perkin Elmer).

### Peptides library synthesis

Pools of 13-mer peptides ([AAA-XXXRme2aXXX-AAA] for antibody characterization, or [KG-XXXXRXXXX-GK] to identify optimal substrate motif for PRMTs) were custom synthesized by Pepscan (Lelystad, Netherlands). The peptide pools were derivatized at their C-termini for covalent attachment to glass slides. Antibody characterization was performed using 13-mer AAA-XXXRme2aXXX-AAA peptides that were arrayed onto glass slides. Six sets of peptides were designed so that the resulting motif spans 3 amino acid residues on either side of a central Rme2a residue (P-3, P-2, P-1 [Rme2a] P+1, P+2, P+3). A single position is fixed in each set of peptides: each set has 19 peptides, wherein one amino acid was fixed at one position, and the rest of the positions have a mixture of the remaining 19 amino acids, except cysteine, in equimolar concentrations to generate a degenerate peptide. Thus, a library of 114 peptides (19 amino acids, 6 positions around central Rme2a) was created. For the PRMT substrate motif screen, a second library was synthesized, this time with a fixed unmodified arginine residue in the central position. The peptide library design of the second library was similar to the first, except that it was larger, with four scanning positions on either side of the fixed arginine residue (KG-XXXXRXXXX-GK). Thus, 152 peptides (19 amino acids, 8 positions around central R) were synthesized. The syntheses of these libraries was performed at a 4 μmol scale. We took 20 μg of each of the 152 peptide pools, and subjected them to Coomassie blue staining (data not shown). We observe roughly equal amounts of peptide in each pool.

### Antibody characterization by the OPAL assay

Each array was incubated with the primary antibody of interest (1:1000 in hybridization buffer [PBS with 5% (w/v) BSA, 0.1% Tween 20]) in a covered microscope slide chamber for 2 hours at 4 °C. The array was then washed, probed with AlexaFlour 647 antibody (1:10,000 in hybridization buffer) for 30 minutes at 4 °C in dark, followed by three washes with PBS (in dark, at 4 °C). Following the last wash, the arrays were air-dried (filtered forced air). Dried arrays were scanned for analysis using Typhoon TRIO+ scanner (GE).

### Quantification of signal for motif

Signal intensities observed in the middle panels of Fig. 3b–d were subject to densitometry analyses using Image J software. This software quantifies a black spot as 0, and white as 255. Each of the eight rows (P-4 to P+4) was handled independently. Intensity values were measured (for each of the 19 peptide libraries at that given position, within each row). For each of the eight rows, the darkest band was taken as 100% and the remaining 18 bands were calculated as a percentage of the darkest band. Each fixed amino acid that had an intensity of 70%, or more, of the darkest band was included in the motif; and depicted as black circles on the array format (top panels of Fig. 3b–d). Amino acids that had intensities in the range of 50–70% of the darkest band are depicted as grey circles. Intensities less than 50% of the darkest band are shown as open circles.

### Recombinant proteins

Recombinant GST-PRMT1 and GST-CARM1 used in the *in vitro* methylation reactions were purified from *E. coli* as previously described^33^. HA-tagged PRMT9 was purified from Sf9 insect cells as previously described^22^,^34^.

### *In vitro* methylation reactions

*In vitro* methylation reactions were performed in 30 μl PBS (pH 7.4) with 1 μg of peptide, 1 μg of recombinant enzymes (PRMT1, CARM1 or PRMT9) and 0.42 μM S-adenosyl-l-[methyl-^3^H]methionine (Perkin Elmer). Reactions were incubated at 30 °C for 1 hour, resolved on 15% SDS-PAGE, transferred to PVDF membrane, treated with En^3^Hance (Perkin Elmer) and exposed to film at −80 °C. Exposure time was different for each PRMT: PRMT1 for 3 days, CARM1 for 2 days, and PRMT9 for 12 days.

## Additional Information

**How to cite this article**: Gayatri, S. *et al*. Using oriented peptide array libraries to evaluate methylarginine-specific antibodies and arginine methyltransferase substrate motifs. *Sci. Rep.* **6**, 28718; doi: 10.1038/srep28718 (2016).
